# Antiviral Activity of Nitrosonium Cations against SARS-CoV-2 on a Syrian Hamster Model

**DOI:** 10.1134/S0006350922050165

**Published:** 2022-12-19

**Authors:** A. V. Shipovalov, A. F. Vanin, O. V. Pyankov, E. G. Bagryanskaya, V. D. Mikoyan, N. A. Tkachev, N. A. Asanbaeva, V. Ya. Popkova

**Affiliations:** 1 State Scientific Research Center of Virology and Biotechnology “Vector”, 630559 Koltsovo, Novosibirsk oblast Russia; 2 Semenov Federal Research Center of Chemical Physics, Russian Academy of Sciences, 119991 Moscow, Russia; 3 Vorozhtsov Institute of Organic Chemistry, Siberian Branch of the Russian Academy of Sciences, 630090 Novosibirsk, Russia

**Keywords:** SARS-CoV-2, binuclear iron dinitrosyl complexes with glutathione, sodium diethyldithiocarbamate, nitrosonium cations

## Abstract

The antiviral action of binuclear dinitrosyl iron complexes with glutathione along with diethyldithiocarbamate against the SARS-CoV-2 virus has been demonstrated on a Syrian hamster model after aerosol exposure of SARS-CoV-2-infected animals to the solutions of said compounds. EPR assays in analogous experiments on intact hamsters have demonstrated that the iron complexes and diethyldithiocarbamate are predominantly localized in lung tissues. These results have been compared with similar measurements on intact mice, which have shown the equal localization of these agents in both the lungs and liver. We assume that the release of the nitrosonium cations from the binuclear dinitrosyl iron complexes with glutathione occurs during their contact with diethyldithiocarbamate in the animal body. These cations caused S-nitrosation of host and viral cell proteases, leading to suppression of SARS-CoV-2 infection.

## INTRODUCTION

Viral infections are characterized by a sharp increase in the level of nitric oxide (NO), one of the universal regulators of metabolic processes in host cells [1–15]. The intracellular increase in the NO concentration is accompanied by S-nitrosation of various viral proteins, i.e., proteases, envelope proteins, reverse transcriptases, transcription factors, and host cell proteases [2–16]. There is reason to assume that this process provides a decrease in viral production, thus being an effective means of protecting the animal and human body from viral infection.

It is known that S-nitrosation of various intracellular components is performed not by NO molecules but by the single-electron oxidized form of these molecules, nitrosonium (NO^+^) cations, which bind instead of protons to thiol groups of thiol-containing proteins and low molecular weight compounds. Therefore, it is necessary to understand the mechanism of conversion of NO to NO^+^ in animal and human cells.

Currently, most researchers believe that this transformation is promoted by the oxidation of NO to nitrogen dioxide, followed by the binding of NO_2_ to NO with the formation of the NO^+^ donor, nitrogen trioxide (N_2_O_3_), capable of S-nitrosating thiols [17–19]. However, it has been shown that the S-nitrosation process can also occur in cell cultures in the absence of oxygen, i.e., without oxidation of NO to NO_2_ [20–22]. Some authors suggest that the NO^+^ cation can appear in animal and human cells due to the formation of dinitrosyl iron complexes (DNICs) with thiol-containing ligands with the participation of NO molecules. The resulting complexes can act as donors of both the NO molecules and nitrosonium cations [20–26].

**Scheme 1.**
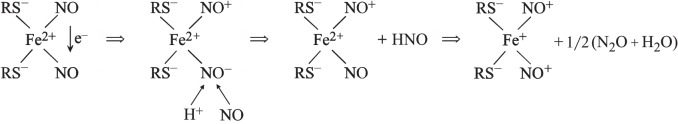
The mechanism for the formation of M-DNIC with thiol-containing ligands in the reaction of Fe^2+^, NO, and thiols. It is assumed that mutual one-electron oxidation-reduction of NO molecules occurs, followed by transformation into nitrosonium cation (NO^+^) and nitroxyl anion (NO^–^) in accordance with this disproportionation reaction [25, 26].

DNICs with thiol-containing ligands were discovered and identified in yeast cells and then in animal tissues by electron paramagnetic resonance (EPR) in the 1960s by one of the authors of this work. This discovery was based on the EPR signal with the average *g*‑factor characteristic of the mononuclear form of DNIC (M-DNIC) (signal with *g*_⊥_ = 2.04, *g*_||_ = 2.014, and *g*_aver_ = 2.03) [27, 28]. In recent years, A.F. Vanin also proposed a mechanism for the formation of these complexes, which is based on the disproportionation of two NO molecules bound to the Fe^2+^ ion in the presence of thiol-containing ligands (Scheme 1).

The nitroxyl anion, which is formed in this reaction as a result of hydrolysis (binding with a proton), turns into the nitroxyl molecule and is released from the complex. Its place is occupied by a neutral NO molecule. The hydrolysis of the remaining nitrosonium cation in the complex (its binding to the hydroxyl anion) is prevented by transferring some of the electron density from thiol sulfur, which is characterized by high π-donor activity. As a result, the positive charge on this nitrosyl ligand is neutralized, and it stops binding to the OH^–^ anions.

Thus, according to the mechanism of the M-DNIC formation (Scheme 1), one of its resonant structures can be represented as [(RS^–^)_2_Fe^2+^(NO)(NO^+^)]^+^. A similar structure may be characteristic of the binuclear form DNIC (B-DNIC), i.e., [(RS^–^)_2_Fe$_{2}^{{2 + }}$(NO)_2_(NO^+^)_2_]^2+^ [25, 26]. The implementation of this structure in the host cells ensures the appearance of donors of nitrosonium, i.e., DNIC with thiol-containing ligands capable of initiating S-nitrosation of various thiol-containing compounds in animal and human cells, thus decreasing and/or excluding viral production in the susceptible cell.

**Scheme 2.**
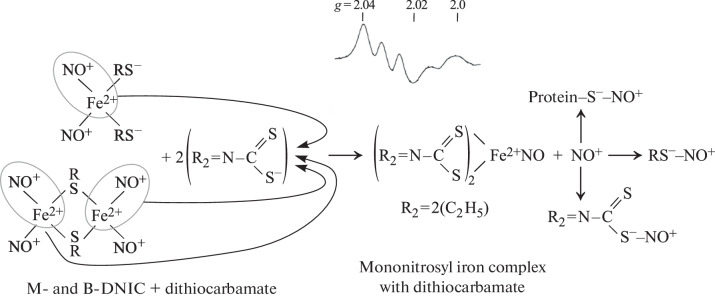
The mechanism of transformation of M-DNIC and B-DNIC with thiol-containing ligands into MNIC with dithiocarbamate derivatives. The released nitrosonium cations can S-nitrosylate low molecular weight thiols, protein thiols, and the thiol group in dithiocarbamate. At the top is the EPR signal recorded at 77 K with *g*-factor values of 2.04 and 2.02 and a triplet hyperfine structure characteristic of MNIC with dithiocarbamate [29, 30].

A.F. Vanin et al. proposed an approach that allows the selective release of the nitrosonium cations from M-DNIC and B-DNiC with simultaneous inclusion of the iron ions and NO molecules into stable complexes, i.e., mononitrosyl iron complexes (MNICs) with dithiocarbamate derivatives, thereby eliminating any influence of the iron ions and NO molecules on intracellular processes. This effect could only be provided by the nitrosonium cations released under the action of dithiocarbamate according to Scheme 2.

In this paper, we studied the antiviral activity of nitrosonium cations against the SARS-CoV-2 virus on Syrian hamsters (an animal model of COVID-19) using B-DNIC with glutathione along with the ditiocarbamate derivative (diethyldithiocarbamate, DETC) as a system capable of delivering nitrosonium cations into the body of these animals by aerosol administration.

## MATERIALS AND METHODS

**Compounds.** We used iron sulfate (FeSO_4_·7H_2_O, Fluka, Schweitzer); reduced glutathione (GSH), N‑acetyl-L-cysteine, sodium diethyldithiocarbamate, and sodium nitrite (Sigma, United States).

**Viruses.** We used the SARS CoV-2 HCoV-19/Russia/Volgda-171613-1208/2020 strain from the State Collection of microorganisms of the SSRC VB “Vector” (Russia). The infectious virus was grown in a *Vero E6* cell culture, and the virus aliquots were frozen and stored at –80°C. The virus titer was at least 10^6^ TCID_50_/mL. Virus of passage 4 was used for the studies. The titer of the viral suspension was evaluated by the method of final dilution on the *Vero E6* cells by the Reed and Muench method [31]. The work with the live virus was carried out under the conditions of the most isolated laboratory, which conform to international requirements BSL3+, in SSRC VB “Vector”, which has permission for these studies.

**Cell cultures.** The *Vero E6* cell line from the collection of the cell cultures of the SSRC VB “Vector” was used in the work. The monolayer of *Vero E6* cells was grown in DMEM medium (Gibco, USA) that contained 10% fetal bovine serum (HyClone, United States) and a complex antibiotic (Gibco, United States). A similar medium that contained 2% fetal bovine serum was used as a supporting medium during virus cultivation.

**Animals.** We used males and females of outbred Syrian hamsters (80–100 g) from the Nursery of Laboratory Animals of the SSRC VB “Vector”. The IPTT-300 transponders (chips) (BMDS, United States) were subcutaneously implanted in hamsters for noncontact temperature measurement and identification. The hamsters were placed (by two animals) in individually ventilated cells. The animals had unlimited access to food and water. Acclimatization to the experimental conditions was carried out for 7 days before infection. The temperature (22–24°C) and relative humidity (40–55%) were maintained during the experiments. Every day, the hamsters were weighed, their temperature was measured, and clinical signs of the disease were assessed.

The optimal concentrations of aerosol-injected compounds were evaluated using males of the CD1 line of outbred mice (18–20 g) from the vivarium of the Emanuel Institute of Biochemical Physics of the Russian Academy of Sciences.

**Model of SARS-CoV-2 infection in Syrian hamsters.** A model of infection of hamsters with the SARS-CoV-2 virus was described earlier [32].

Six groups of animals (two experimental and one control) were used with eight hamsters (four males and four females) in each. Before infection, hamsters were anesthetized with an intramuscular injection of Zoletil 100 (Virbac, France). Anesthetized animals were infected intranasally by inoculation of the virus with a pipette in a volume of 50 μL at a dose of 50 ID_50_. After infection for 120 h, all animals were subjected to euthanasia by dislocation of the cervical vertebrae. An autopsy was performed, and the tissues of the nasal passages and lungs were taken. A mechanical homogenizer (FastPrep-24, MP Biomedical, United States) was used to obtain 10% tissue homogenates, which were clarified by centrifugation at 10 000 rpm (rotor SW28, Beckman Coulter, United States). The amount of viral RNA in aliquots of the clarified samples was evaluated by real-time reverse transcription polymerase chain reaction (RT-PCR) through a surrogate *C*t indicator (number of cycles). The concentration of the infectious virus was evaluated in terms of TCID_50_/mL by titration in the *Vero E6* cell culture.

**Evaluation of RNA of SARS-CoV-2 virus in biological samples by real-time RT-PCR.** We used the RIB-prep kit (AmpliSens, Russia) to isolate RNA. The cDNA synthesis from isolated RNA was carried out using reagents for the reverse transcription reaction Reverta-L (Central Research Institute of Epidemiology, Moscow). Amplification of cDNA fragments of the SARS-CoV-2 virus was performed using the reagents of the PCRRV-COVID19-RG kit (SSCR VB “Vector”, Russia). The results of the study were analyzed according to the manufacturer’s protocol.

**Evaluation of the infectious titer of the SARS-CoV-2 virus in biological samples on a** ***Vero E6*** **transferable cell culture.** *Vero E6* cells were seeded 24 h before infection into 96-well plates with a sowing dose of 1.5 × 10^4^ cells/well. On the day of the experiment, sequential tenfold dilutions of the virus were performed using DMEM medium (Gibco, United States) containing 2% of fetal bovine serum (HyClone, United States) and a complex antibiotic (Gibco, United States). In total, the cells in 6–8 wells were infected with each dilution of the virus. After incubation for 72 h, the cells were fixed with a 4% solution of buffered formalin, followed by staining with 0.1% crystal violet. The specific lesion of the monolayer of the cell culture in the well was taken into account as TCID. The virus titer was calculated using the Reed-Muench formula [31] and expressed in log TCID_50_/mL.

**Injection of compounds.** Aerosol injection of compounds was carried out in a vertical dynamic chamber for 30 min. The working airflow through the chamber was 10 L/min. Eight hamsters were simultaneously placed in the inner volume of the chamber. The dispersion was carried out with an Omron nebulizer with an airflow rate of 6 L/min. 10 mL of the 10 mM aqueous solution of the preparations was placed into the nebulizer. The selected aerosol was captured using the MC-2 sampler connected to the outlet fitting of the chamber. The volume sampling rate of the aerosol in the sampler was 10.0 ± 0.5 L/min, and the volume of the liquid (distilled water) was 10 mL.

Similarly, the same compounds were introduced to CD 1 outbred mice not infected with the coronavirus.

The administration of the drug to the infected Syrian hamsters was carried out for four days twice a day (the first administration was 1 h after infection). The animals were euthanized 120 h after infection (by dislocation of the cervical vertebrae); an autopsy was performed, and the tissues of the nasal passages and lungs were taken (under aseptic conditions) to evaluate the amount of virus RNA in tissue homogenates by real-time RT-PCR through the *C*t value (the number of cycles) and by titration on *Vero 6* cell culture (logTCID_50_/mL).

**Scheme 3.**
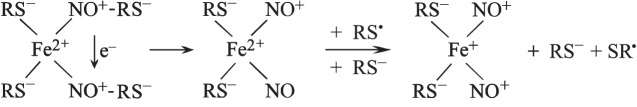
Mechanism of M-DNIC formation in the reaction of S-nitrosothiols, Fe^2+^, and thiols [33].

**Synthesis of binuclear dinitrosyl iron complexes with glutathione.** To synthesize B-DNIC with glutathione (B-DNIC-GSH), we used the ability of S-nitrosothiols to form the corresponding M-DNIC in reaction with Fe^2+^ ions and thiols (Scheme 3).

**Scheme 4.**
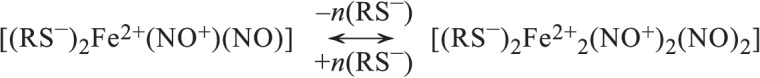
Equilibrium interconversion of M-DNIC and B-DNIC with thiol-containing ligands. The equilibrium shifts to the left when the level of thiols (RS^–^) ionized by the thiol group increases in the solution [34].

**Fig. 1.**
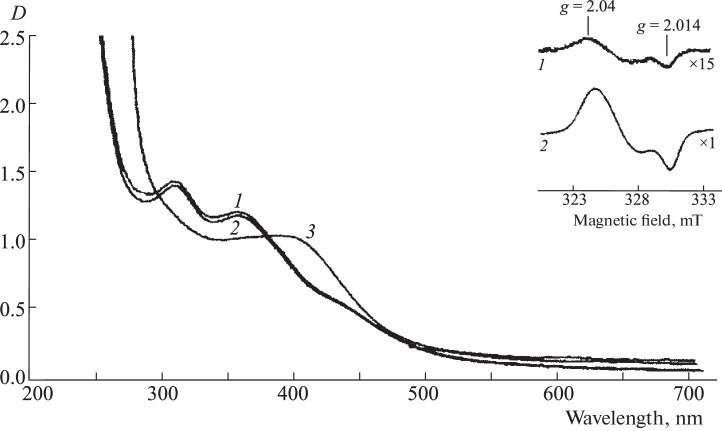
Optical absorption spectra of B-DNIC-GSH and M-DNIC-GSH at a concentration of 0.46 mM (spectra 1 and 3, respectively); on the insert is the EPR signal of M-DNIC-GSH (signal 2.03 with *g*-factor tensor values of *g*_⊥_ = 2.04 and *g*_||_ = 2.014) [25].

Fe^2+^ ions bind two S-nitrosothiol molecules per ion, followed by disproportionation of these molecules, thus leading to the immediate formation of M‑DNICs, which is characterized, as mentioned above, by an EPR signal with *g*_aver_ = 2.03 (signal 2.03). At a low concentration of thiols in the solution, M-DNICs are condensed into diamagnetic B-DNIC complexes (Scheme 4), which do not give the signal 2.03. Their concentration could be estimated by the intensity of two optical absorption bands at 310 and 360 nm (Fig. 1) with molar absorption coefficients of 4600 and 3700 M^–1^ cm^–1^, respectively, per one atom of iron in B-DNIC.

The synthesis of B-DNIC-GSH (the 10 mM solution) was carried out accordingly [35]. Glutathione, ferrosulfate, and sodium nitrite at concentrations of 40, 20, and 20 mM, respectively, were sequentially introduced into a 15 mM HEPES buffer solution, pH 7.2–7.4. The mixture was thoroughly stirred after each addition. The introduction of glutathione led to acidification of the buffer solution to pH 3.6–3.8. Before the subsequent introduction of ferrosulfate, the pH value of the solution was lowered with sulfuric acid to pH 1–2 to prevent the formation of iron hydroxide complexes. This pH value also guaranteed the more efficient formation of S-nitrosoglutathione after the addition of sodium nitrite to the reaction mixture. This process was monitored by the intensity of the absorption band at 334 nm with ε = 0.94 M^–1^ cm^–1^, which is characteristic of S-nitrosoglutathione [36]. Usually, the formation of 20 mM S-nitrosoglutathione is completed at room temperature for 1.5 h. After that, the pH of the solution, which is colored pink due to the presence of S-nitrosoglutathione, was increased to pH 7.3–7.5 by dropwise addition of a strong (100 mM) NaOH solution, which led to the change of the color of the solution to orange due to the formation of the M-DNIC-GSH complex (Scheme 3). The solution was left overnight in the air at room temperature. During this time, half of the iron (10 mM) was included in M-DNIC-GSH and then in B-DNIC-GSH, while the other half of the iron was included in the precipitated water-insoluble hydroxide complexes (Schemes 3 and 4). The next day, this precipitate was removed by filtering the solution through a paper filter.

A clear 10 mM solution of B-DNIC-GSH was obtained, which was frozen in liquid nitrogen and stored in a frozen state at a temperature no higher than –18°C before use in animal experiments. Figure 1 shows the absorption spectra of 0.5 M B-DNIC-GSH (spectrum 1) with absorption bands at 310 and 360 nm. The spectrum did not change for an hour when the solution was acidified to pH 1.0 (spectrum 2). The insert shows the EPR signal of the 10 mM solution of B-DNIC-GSH (signal 2.03, spectrum 1), which is caused by a 5% impurity of M-DNIC. When 10 mM GSH was added to this solution, the pH value increased from 7.5 to 10.5, and B-DNIC was completely converted into M-DNIC. The absorption spectrum of the latter with the maximum at 400 nm (Fig. 1, curve 3) and the EPR signal 2.03 of M-DNIC (the insert) are shown in Fig. 1

**Recording EPR spectra of isolated tissues of native (uninfected) animals.** After the experiment, uninfected animals (Syrian hamsters and mice) were euthanized by dislocation of the cervical vertebrae for subsequent evaluation of the localization of B-DNIC and M‑DNIC-DETC in isolated blood samples and tissues of the lungs and liver of animals by EPR. Blood and organ tissues were taken under aseptic conditions. The samples (lungs, liver, and blood) were placed in plastic tubes with a diameter of 4 mm and frozen in liquid nitrogen. For subsequent recording the EPR spectra, tubes with frozen tissues were removed from liquid nitrogen, and the samples were gradually heated in the hands, squeezed out into liquid nitrogen with a piston, and placed in liquid nitrogen in a Dewar finger of the appropriate shape. The EPR spectra of the samples were recorded with the appropriate parameters of an EPR radio spectrometer (Bruker, Germany) or a modified EPR radio spectrometer (RadioPan, Poland). A solution of M-DNIC-GSH was used as a reference sample (EPR signal 2 in Fig. 1) to evaluate the concentration of M-DNIC in animal tissues. The content of M-DNIC in the lung tissue, which was assessed by the standard, was doubled because the density of the lung tissue is about half the density of water.

## RESULTS

**Evaluation of the effectiveness of the compounds B‑DNIC-GSH and DETC with aerosol administration.** The analysis of the results of RT-PCR (*C*t) (Table 1) shows that the treatment of the animals with only the B-DNIC-GSH solution leads to a significant decrease in the viral load in the tissues of the nasal cavity by a factor of 16 (*p* = 0.0006 according to the Mann-Whitney criterion) compared with that in the control animals (placebo). At the same time, according to the infectious titer (logTCID_50_/mL), no significant decrease in the viral load was recorded in either the tissues of the nasal cavity or the lung tissues (*p* > 0.005 according to the Mann-Whitney criterion).

**Table 1.** Viral load in the nasal cavity and lung tissues of hamsters intranasally infected with the SARS-CoV-2 virus, followed by aerosol treatment with B-DNIC-GSH and B-DNIC-GSH + DETC

Compound	Viral load SARS-CoV-2			
	in the tissues of the nasal cavity		in the lung tissues	
	logTCID_50_/vk	Ct*	logTCID_50_/ml	Ct*
B-DNIC-GSH	4.1 ± 0.5	23.83 ± 1.27	4.4 ± 0.8	20.67 ± 2.13
B-DNIC-GSH + DETC	2.7 ± 0.4	27.28 ± 3.44	2.4 ± 0.4	30.33 ± 1.18
Placebo	5.0 ± 0.5	19.80 ± 2.19	3.7 ± 0.4	25.96 ± 1.60

* Lower threshold values of the number of cycles (Ct) indicate higher viral loads.

The reliable antiviral activity was evaluated for the sequential administration of the B-DNIC-GSH + DETC system. According to the RT-PCR (*C*t), the level of RNA accumulation in the lung and the nasal cavity tissues decreased by factors of 21 and 16 (*p* = 0.0007 and 0.0025 according to the Mann-Whitney criterion), respectively. The titration method in the tissues of the nasal cavity showed an even greater (200-fold) decrease in viral load (*p* = 0.0002). A 20-fold decrease in viral load in the lung tissues was observed (*p* = 0.0002 according to the Mann-Whitney criterion).

The proposed sequence of administration of B‑DNIC-GSH and DETC into the respiratory tract of hamsters should lead to the release of the nitrosonium cations from B-DNIC and their accumulation in the tissues, thus providing suppression of SARS-CoV-2 infection. The results of aerosol administration of DETC or B-DNIC-GSH after DETC led to no significant decrease in the viral load in sensitive tissues of target organs for two the studied parameters compared to control animals (placebo) (Table 2).

**Table 2.** Viral load in the nasal cavity and lung tissues of hamsters intranasally infected with the SARS-CoV-2 virus, followed by aerosol treatment with DETC and DETC + B-DNIC-GSH

Compound	Viral load SARS-CoV-2			
	in the tissues of the nasal cavity		in the lung tissues	
	logTCID_50_/vk	Ct*	logTCID_50_/ml	Ct*
DETC	5.3 ± 0	11.59 ± 2.04	5.5 ± 0	13.31 ± 1.28
DETC + B-DNIC-GSH	5.3 ± 0	10.78 ± 0.44	5.2 ± 0.3	14.60 ± 0.86
Placebo	5.5 ± 0	9.60 ± 2.02	4.3 ± 0.1	15.54 ± 1.84

* Lower threshold values of the number of cycles (Ct) indicate higher viral loads.

**Fig. 2.**
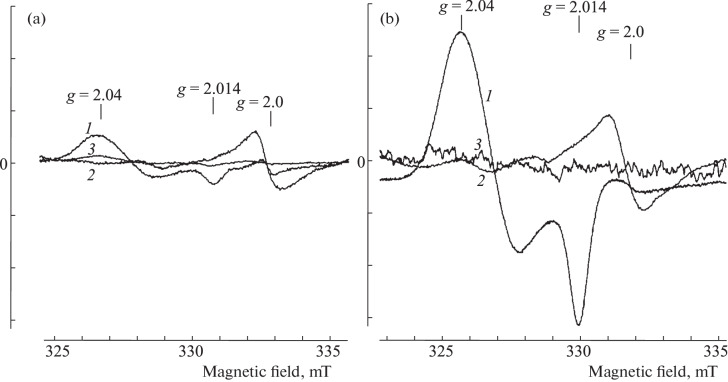
EPR spectra of the lungs (*1*), liver (*2*), and blood (*3*) of hamsters exposed to inhalation of a sprayed 10 mM solution of DNIC (a) and 10 mM solutions of DNIC, followed by DETC (DNIC + DETC) (b). The signal at *g* = 2.0 is due to endogenous free radicals. The spectra were recorded on a Bruker radio spectrometer at 77 K, microwave power of 5 MW, amplitude of high-frequency modulation of the magnetic field of 0.5 mT, and the same gain of the radio spectrometer.

**EPR measurement of the tissues of Syrian hamsters.** One of the typical EPR spectra recorded at 77 K in the blood, lungs, and liver of healthy (uninfected) hamsters after aerosol administration of 10 mM solution of B-DNIC-GSH (10 mL) for 30 min is shown in Fig. 2a. The EPR spectra of the same hamster tissues after aerosol administration of a 10 mM solution of B‑DNIC-GSH (10 mL), followed by a 30-min injection of a 10 mM DETC solution (DNIC – + DETC) is shown in Fig. 2b. In both cases, the highest signal 2.03 (EPR signal appearing in the tissues of M‑DNIC) was observed in the lungs. When animals inhaled only the B-DNIC-GSH solution, the M‑DNIC concentration in this organ varied within 30–40 μmol/kg of wet tissue. In the blood, this value varied in the range of 3‒5 μmol/L, and signal 2.03 was almost not detected in the liver. When animals sequentially inhaled solutions of B-DNIC-GSH and DETC (DNIC + DETC), the level of M-DNIC in the lungs reached 140–160 μmol/kg based on the intensity of signal 2.03. In other words, the average level of M‑DNIC increased four to five times compared to the value observed when the animals were treated only with B‑DNIC-GSH (Fig. 2a). In the liver, the M‑DNC content reached 3 μmol/kg; the signal 2.03 was not detected in the blood (Fig. 2b).

**Fig. 3.**
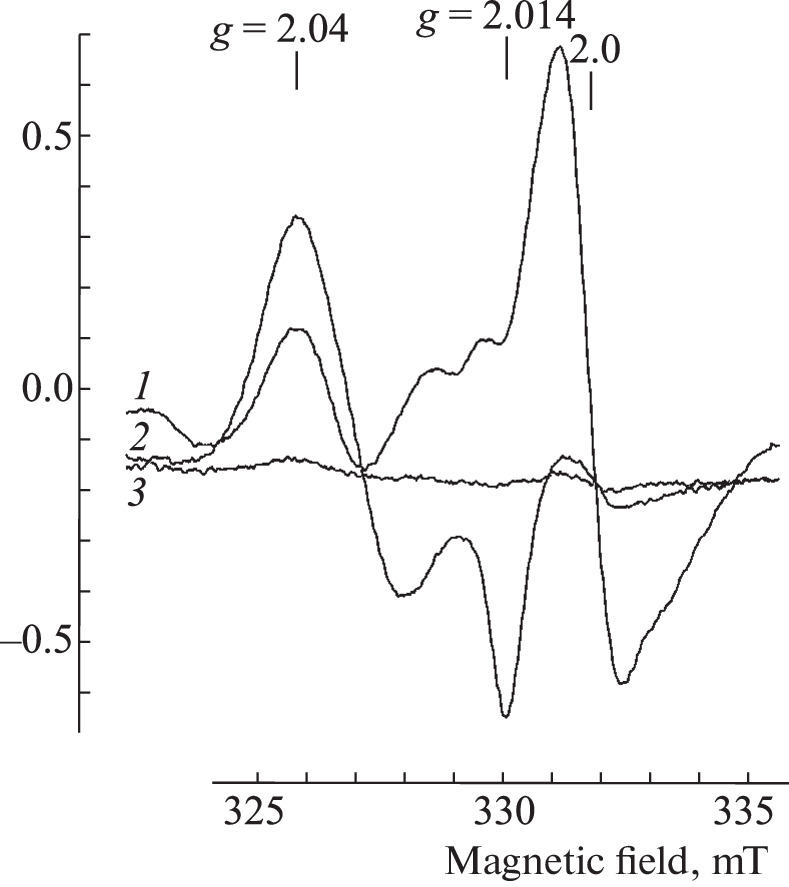
EPR spectra of lungs (*1*), liver (*2*), and blood (*3*) of hamsters exposed to 30-min inhalation of a sprayed 10 mM DETC solution followed by 30-min inhalation of a sprayed 10 mM solution of B-DNIC-GSH (DETC + DNIC). The signal at *g* = 2.0 is caused by endogenous free radicals. The spectra are recorded on a Bruker radio spectrometer at 77 K, microwave power of 5 MW, RF modulation amplitude of the magnetic field of 0.5 mT, and the same gain of the radio spectrometer.

Figure 3 shows the EPR spectra recorded in the blood, lungs, and liver of hamsters after aerosol treatment with the 10 mM DETC solution for 30 min, followed by inhalation of the 10 mM solution of B‑DNIC-GSH. Judging by the signal intensity of 2.03 in these tissues, the highest amount of M-DNIC was found in the lungs (30–40 μmol/kg). The maximum concentration in the liver and the blood reached 10 μmol/kg and 0.5 μmol/kg, respectively.

**Fig. 4.**
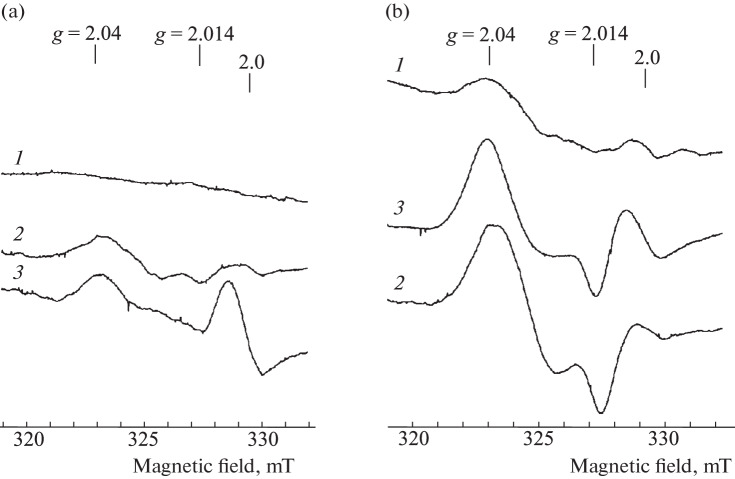
Signal 2.03 in the blood (*1*), lungs (*2*), and liver (*3*) of mice subjected to 40-min inhalation of 10 mM solution of B-DNIC-GSH (a), followed by 40-min inhalation of 50 mM solution of DETC (b). The signal at *g* = 2.0 is caused by endogenous free radicals. The spectra were recorded on a RadioPan radio spectrometer at 77 K, microwave power of 5 MW, the amplitude of the RF modulation of the magnetic field of 0.5 mT, and the same gain of the radio spectrometer.

**EPR measurement of the mice tissues.** Aerosol administration of the 10 mM B-DNIC-GSH solution to mice (10 mL) for 40 min led, judging by the signal intensity of 2.03, to the appearance of M-DNIC in the lungs and liver at concentrations of 0.5–1.2 and 1.5–2.0 μmol/kg, respectively (Fig. 4a). In contrast to Syrian hamsters, B-DNIC-GSH after inhalation was quite effectively transferred from the lungs to the blood and then to the liver. Nevertheless, only a very weak signal 2.03 was recorded in the blood. After the subsequent aerosol administration of a 10 mM solution of DETC (10 mL) to these animals, the level of M‑DNIC in the tissues of mice almost did not increase. With an increase in the DETC concentration up to 50 mM, the concentration of M-DNIC in all the studied tissues significantly increased (by factors of 3–4) (Fig. 4b). At the same time, the level of M-DNIC in the lungs, liver, and blood varied within 1.5–3.0, 0.8–1.4, and 0.3–0.5 μmol/L, respectively.

**Fig. 5.**
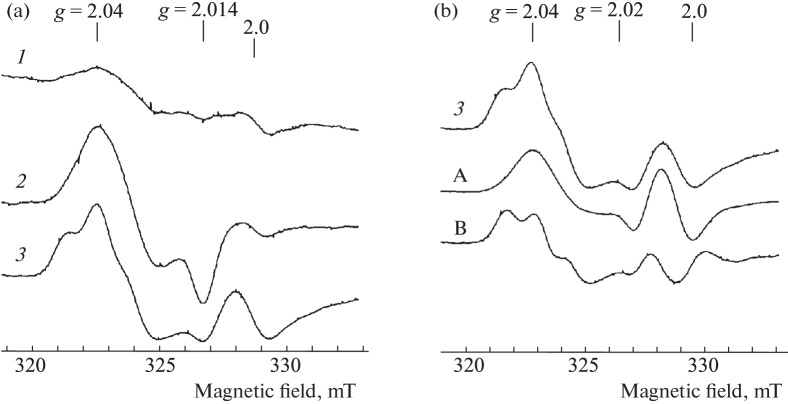
(a) EPR spectra recorded in the blood (*1*), lungs (*2*), and liver (*3*) of mice subjected to 40-min inhalation of a sprayed 50 mM solution of DETC, followed by inhalation of a 10 mM solution of B-DNIC-GSH. (b) EPR spectra recorded in the liver (*3*) as the sum of signal 2.03 (A) and the MNIC-DETC (B) signal. The signal at *g* = 2.0 is caused by endogenous free radicals. The spectra were recorded on a RadioPan radio spectrometer at 77 K, microwave power of 5 MW, the amplitude of the RF modulation of the magnetic field of 0.5 mT, and the same gain of the radio spectrometer.

With the reverse sequence of aerosol administration to mice (50 mM of DETC solution, followed by 10 mM of B-DNIC-GSH solution), the ratio of the content of M-DNIC in the tissues of mice did not change (Fig. 5a), i.e., it remained highest in the lungs (6–10 μmol/kg), reached 0.8 μmol/L) in the blood and 1.5–3.0 μmol/kg in the liver (Fig. 5). At the same time, along with the signal 2.03, we observed the EPR signal of MNIC-DETC complexes in the liver due to the interaction of DETC with DNIC (according to Scheme 2). The EPR signal of these complexes was characterized (in accordance with [37]) by the *g* factor values of *g*_⊥_ = 2.04, *g*_||_ = 2.02, *g*_aver_ = 2.033, and the resolved triplet hyperfine structure at *g*_⊥_ (Fig. 5b).

**Fig. 6.**
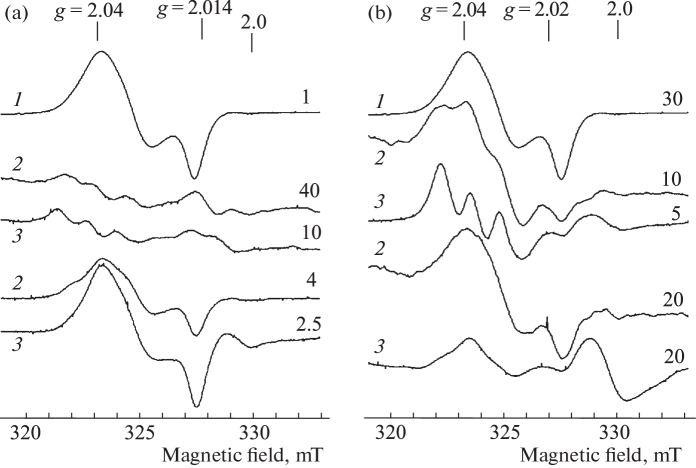
EPR spectra recorded in the blood (*1*), lungs (*2*), and liver (*3*) of mice. (a) Animals were exposed to 40-min inhalation of 50 mM sprayed solution of DETC (spectra 40 and 10), followed by i/p administration of B-DNIC-GSH at a dose of 100 μmol/kg of animal weight (spectra 4, 2.5, and 1). (b) Animals were exposed to 40-min inhalation of of 10 mM with a sprayed solution of B-DNIC-GSH (spectra 30, 20, and 20), followed by the administration of DETC at a dose of 0.5 mM/kg of animal weight (spectra 10 and 5). The signal at *g* = 2.0 is caused by endogenous free radicals. The spectra were recorded on a RadioPan radio spectrometer at 77 K, microwave power of 5 MW, the amplitude of the RF modulation of the magnetic field of 0.5 mT, and the same gain of the radio spectrometer.

The EPR signal of MNIC-DETC in its pure form was detected in the lungs after inhalation of only 50 mM DETC solution (Fig. 6a). The concentration of MNIC did not exceed 0.4 μmol/kg. After subsequent intraperitoneal (i/p) administration of B-DNIC-GSH to these animals at a dose of 100 μmol/kg of animal weight, the MNIC-DETC signal in the lungs was masked by the 2.03 signal because of an admixture of blood (Fig. 6a). The M-DNIC concentration in the blood of these animals reached 30 μmol/L (Fig. 6a). As in the lungs, the EPR signal of MNIC-DETC in its pure form in the liver was detected after inhalation of animals with only 50 mM DETC solution, while the concentration of these complexes reached 3.0 μmol/kg (Fig. 6a). As in the lungs, the MNIC-DETC signal in the liver was masked by the 2.03 signal after intraperitoneal administration of B-DNIC-GSH to mice at a dose of 100 μmol/kg (Fig. 6a).

Noticeable EPR signals of MNIC-DETC in the lungs and liver were recorded after aerosol administration of 10 mM solution of B-DNIC-GSH to mice, followed by intraperitoneal administration of DETC at a dose of 500 μmol/kg of animal weight (Fig. 6b). In the lungs, signal 2.03 was superimposed on this signal, so that the sum concentration of MNIC-DETC and M‑DNIC in this tissue was 5.0 μmol/kg, provided that the concentration of M-DNIC after the initial administration of B-DNIC-GSH was 1 μmol/kg (Fig. 6b). The concentration of MNIC-DETC in the liver reached 10 μmol/kg of tissue, provided that the concentration of M-DNIC after the initial aerosol administration of B-DNIC-GSH to mice was 0.6 μmol/kg (Fig. 6b). The formation of MNIC-DETC was not detected in the blood in similar experiments, i.e., after i/p administration of DETC, the M-DNIC concentration remained at the level of 0.5 μmol/L, which appeared in the blood after inhalation of B-DNIC-GSH (Fig. 6b).

A significant difference in the concentration of the formed MNIC-DETC and M-DNIC in the liver (as in the lungs) was obviously caused by the inclusion of DNIC in these tissues mainly in the binuclear EPR-inactive form of these complexes. The same conclusion follows from experiments when the N-acetyl-L-cysteine solution was i/p injected into mice at a dose of 100 μmol/kg at neutral pH values after aerosol administration of the B-DNIC-GSH solution. Both in the lungs and liver of the animals, the intensity of signal 2.03 doubled after this treatment (following Scheme 2). The inclusion of N-acetyl-L-cysteine in these tissues ensured the conversion of some of the B-DNIC into M-DNIC. The weaker effect of N-acetyl-L-cysteine compared to the effect of DETC on B-DNIC was caused, in our opinion, by the high affinity of DETC to the iron-mononitrosyl group, which ensured almost complete conversion of B-DNIC into MNIC-DETC.

## DISCUSSION

It was shown that the combination of the compounds B-DNIC-GSH + DETC has a pronounced antiviral effect against the SARS-CoV-2 virus. According to Scheme 2, the proposed order of the introduction of these compounds into the respiratory tract of animals should lead to the release of the nitrosonium cations from B-DNIC and their accumulation in the tissues of the respiratory tract and lungs of hamsters. The nitrosonium cations could suppress COVID-19 infections according to the hypothesis expressed by A.F. Vanin [16].

The EPR study of healthy hamsters and mice exposed to inhalation of sprayed solutions of B‑DNIC-GSH and DETC showed that the appearance of the nitrosonium ions in the tissues of target organs depended on the order of the addition of the proposed compounds. After inhalation of B-DNIC-GSH, followed by DETC, a noticeable amount of nitrosonium cations can accumulate in the tissues of the respiratory tract and lungs, which are targets of the SARS-CoV-2 virus.

An unexpected result of our studies is that the above order of administration of these compounds to healthy hamsters did not lead to the formation of EPR-active MNIC-DETC in the lungs (Fig. 2) as predicted by Scheme 2. The only detected effect after the sequential inhalation of B-DNIC-GSH and DETC was a significant (3–4-fold) increase in the intensity of the 2.03 signal recorded in the lungs (Fig. 2a, b). If we take into account that the injected B-DNIC-GSH complexes are EPR-inactive and that the signal 2.03 is caused by the mononuclear form of DNIC, the above-mentioned amplification of this signal may be due to the appearance in the lung tissue of an additional amount of thiols, which promote the transformation of DNIC into M-DNIC according to Scheme 4. This means that DETC, as the thiol-containing agent can increase the intracellular reduction potential in the tissues, thereby ensuring the reduction of disulfides to thiols.

Another, apparently more realistic mechanism of increasing the level of reducing agents (or decreasing intracellular oxidative potential) may be the following. The reaction of DETC with DNIC can lead to the formation of the MNIC-DETC complex (Scheme 2), which can actively bind superoxide anions, thus neutralizing their prooxidant effect [38]. It is known that the lungs are in contact with air oxygen. Therefore, it can be expected that the presence of a significant amount of superoxide anions in the lung tissues is sufficient for the decay of MNIC-DETC when these complexes bind to superoxide anions. This decay can be caused, for example, by the isomerization of peroxynitrite into nitrate when peroxynitrite is formed by the binding of superoxide to the NO molecule in the ligand sphere of iron in MNIC-DETC [38]. In addition, MNIC-DETC-bound peroxynitrite can oxidize DETC to its dimeric disulfide form (disulphiram) under the catalytic action of iron, which should also lead to the disintegration of these complexes. This kind of decay of MNIC-DETC could lead to the disintegration of all these complexes, which could be the reason for our failure to record the transformation of B-DNIC-GSH after the sequential aerosol injection of the B-DNIC and DETC solutions to Syrian hamsters. In this case, the nitrosonium cation should be released from the B-DNIC according to Scheme 2 and demonstrate an antiviral effect, which could be evaluated by the viral load in the tissues of the target organs of Syrian hamsters.

As a result of the action of the aerosol solution of DETC, the formation of MNIC-DETC was detected in the lungs and liver of mice (Fig. 6a), and the concentration of these compounds in the liver was an order of magnitude higher than in the lungs. The appearance of these complexes was most likely due to the generation of endogenous NO in the lungs and liver, which, together with weakly bound intracellular (free) iron and DETC, forms corresponding MNICs. A significant amount of the superoxide anions should obviously have been produced in the lungs in contact with atmospheric oxygen. Therefore, the action of these anions, as indicated above, should have led, firstly, to the disintegration of a significant part (significantly larger than in the liver) of the MNIC-DETC complexes and, secondly, to the disappearance of DETC entering the lungs. During the subsequent (after DETС) aerosol treatment of animals with the B‑DNIС solution, the reaction of these complexes with DETC became impossible; the MNIC-DETC complexes were not formed and could not provide a decrease in the level of superoxide anions and, consequently, an increase in intracellular reduction potential. This could lead to the fact that an increase in the number of EPR-active M-DNIC was not detected, judging by signal 2.03, as was in the case when treating hamsters sequentially with a sprayed solution of B‑DNIC-GSH and DETC (Fig. 2 and 3). The concentration of M-DNIC in the lungs of hamsters after sequential inhalation of the DETC and B-DNIC-GSH solutions (Fig. 3) was the same as in experiments with inhalation of only the B-DNKIC-GSH solution (Fig. 2a).

Aerosol administration of B-DNIC-GSH in the animal body led to the accumulation of M-DNIC mainly in the lungs of the Syrian hamsters and in the lungs and the liver of mice at similar concentrations. This phenomenon was obviously caused by the weakening of the hemato-tissue barrier in mice, i.e., easier penetration of various substances (both B-DNIC and DETC) from cells into the blood and vice versa. This led to the fact that after sequential administration of DETC and B-DNIC-GSH, both substances could form EPR-recorded MNIC-DETC in the liver (Fig. 5). A more significant amount of these complexes were detected in the liver if B-DNIC-GSH was administered to mice intraperitoneally after aerosol administration of DETC (Fig. 6b). Interestingly, the level of MNIC-DETC in the liver was higher than in the lungs even with this method of administration of compounds.

In conclusion, the results of our previous work [39] should be noted , which showed that aerosol administration of B-DNIC-GSH in rats led to the predominant localization of M-DNIC in lung tissues as in the case of Syrian hamsters. It can be assumed that the possible use of the B-DNIC-GSH + DETC scheme for the treatment of COVID-19 in humans can lead to the appearance of the nitrosonium cations during the decay of DNIC predominantly in the tissues of the lungs and respiratory tract, thus exhibiting a local antiviral effect.
